# Does Adjusted Global Antiphospholipid Syndrome Score (aGAPSS) Predict the Obstetric Outcome in Antiphospholipid Antibody Carriers? A Single-Center Study

**DOI:** 10.1007/s12016-021-08915-9

**Published:** 2021-12-15

**Authors:** Sara Del Barrio-Longarela, Víctor M. Martínez-Taboada, Pedro Blanco-Olavarri, Ana Merino, Leyre Riancho-Zarrabeitia, Alejandra Comins-Boo, Marcos López-Hoyos, José L. Hernández

**Affiliations:** 1 Division of Rheumatology, Hospital Marqués de Valdecilla-IDIVAL, Santander, Spain; 2 Division of Obstetrics and Ginecology, Hospital Marqués de Valdecilla, Santander, Spain; 3 Rheumatology Department, Hospital Sierrallana-IDIVAL, Torrelavega, Spain; 4 Immunology Department, Hospital Universitario Marqués de Valdecilla-IDIVAL, Santander, Spain; 5 Department of Internal Medicine, Hospital Marqués de Valdecilla-IDIVAL, Santander, Spain; 6 University of Cantabria, Santander, Spain

**Keywords:** Pregnancy; Antiphospholipid syndrome; Antiphospholipid antibodies, GAPSS, Score, Non-criteria obstetric manifestations

## Abstract

The adjusted Global Antiphospholipid Syndrome (APS) Score (aGAPSS) is a tool proposed to quantify the risk for antiphospholipid antibody (aPL)-related clinical manifestations. However, aGAPSS has been validated mainly for thrombotic events and studies on APS-related obstetric manifestations are scarce. Furthermore, the majority of them included patients with positive aPL and different autoimmune diseases. Here, we assess the utility of aGAPSS to predict the response to treatment in aPL carriers without other autoimmune disorders. One-hundred and thirty-seven women with aPL ever pregnant were included. Sixty-five meet the APS classification criteria, 61 had APS-related obstetric manifestations, and 11 were asymptomatic carriers. The patients’ aGAPSS risk was grouped as low (< 6, *N* = 73), medium (6–11, *N* = 40), and high risk (≥ 12, *N* = 24). Since vascular risk factors included in the aGAPSS were infrequent in this population (< 10%), the aGAPSS score was mainly determined by the aPL profile. Overall, the live birth rate was 75%, and 37.2% of the patients had at least one adverse pregnancy outcome (APO). When considering patients according to the aGAPSS (high, medium, and low risk), no significant differences were found for pregnancy loss (29.2%, 25%, and 21.9%) or APO (33.3%, 47.5%, and 32.9%). In the present study, including aPL carriers without other autoimmune diseases, aGAPSS is not a valuable tool to identify patients at risk for obstetric complications despite treatment. In these patients with gestational desire, in addition to the aPL profile, other pregnancy-specific factors, such as age or previous obstetric history, should be considered.

## Introduction

Systemic autoimmune diseases mostly affect women during their childbearing years [1, 2]. Some of them, especially systemic lupus erythematosus (SLE) and antiphospholipid syndrome (APS), are associated with a poor obstetric outcome with an increase in pregnancy losses and obstetric complications, including preeclampsia and premature birth [3–5].

The autoantibody profile in these patients plays a clear role in the obstetric outcome [6], and several studies have suggested that antiphospholipid antibodies (aPLs) are the key elements in predicting the risk of developing complications during future pregnancies [3, 7–9]. In this regard, during the last few years, three main groups of researchers have developed scores including the main aPLs in an attempt to stratify the risk of patients carrying these antibodies [10–12]. The APL-score [12], the GAPPS score [11], and more recently, the EUREKA algorithm [10] have attempted to stratify the risk of developing clinical manifestations in patients with aPL. Whereas the first two scores have been mainly validated in APS with thrombotic manifestations [13–19], the EUREKA algorithm has been developed mainly for obstetric manifestations and has not yet been validated in external cohorts [10]. Furthermore, most of the studies that analyzed the impact of these scores on pregnancy outcomes have been done in cohorts that mostly included patients with various autoimmune diseases, especially SLE [13, 20, 21]. Finally, since the GAPSS score and the APL-score include laboratory parameters not routinely performed in daily clinical practice, such as anti-phosphatidylserine/prothrombin antibodies, both scores have implemented variants adapted to the clinically available aPLs [22, 23].

Taking into account these considerations, our study aimed to analyze the role of aGAPSS, the most widely used score in the literature, in a cohort of pregnant women with aPL without other associated diseases. The ability of aGAPSS to predict the response to medical treatment in subjects with aPL was evaluated not only concerning pregnancy loss but also the development of serious obstetric complications during pregnancy. Furthermore, a literature review was carried out on the main scores to evaluate obstetric APS.

## Subjects and Methods

### Study Participants

This retrospective study included 137 consecutive ever pregnant women with confirmed aPL according to the Sidney classification criteria [24]. All of them were followed at the Autoimmune Diseases Pregnancy Clinic, a multidisciplinary unit of a teaching tertiary care hospital, between 2005 and March 2021. The information collected from individual cases was completely anonymized, and the study was approved by the Ethics Committee of Cantabria (internal code: 2021.037).

The inclusion criteria were the following ones: (a) ever pregnant women with confirmed aPL positivity (according to Sidney criteria [24]) and (b) women who received treatment according to the standard of care during pregnancy [25–28]. Women who fulfilled the classification criteria for rheumatic autoimmune diseases other than APS were excluded.

### Data Collection

Data were collected using a prespecified standardized questionnaire in a computerizing database.

We assessed the following clinical variables:

- Demographic and General Characteristics of the Study Cohort: age, sex, body mass index (BMI), current/past tobacco use, high blood pressure (equal or greater than 140/90 mm Hg or being on antihypertensive agents), dyslipidemia (serum total cholesterol or triglyceride levels greater than 230 mg/dl and 150 mg/dl respectively or being on lipid-lowering drugs), diabetes mellitus (according to the ADA criteria), past or present family (< 50 years), or personal history of thrombotic disease.
- Comorbidities: the three main entities associated with pregnancy outcomes were also recorded: (a) inherited thrombophilia (factor V Leiden, prothrombin mutation, protein S and/or C deficiency), (b) thyroid disease (history of hypo/hyperthyroidism or the presence of confirmed specific autoantibodies), and (c) obstetric comorbidity (local uterine abnormalities, endometriosis, and polycystic ovary syndrome).
- Autoantibody Detection: the presence of the following antibodies and isotypes of aPL was quantified by commercial enzyme immunoassay in solid phase (ELISA; Orgentec Diagnostika GmbH, Mainz, Germany): anticardiolipin antibodies (aCL) of the IgG and IgM isotype and anti-beta2 glycoprotein I antibodies (AB2GPI) of the IgG and IgM isotype. The results are reported as quantitative and semiquantitative values. Thus, aCL were quantified in GPL (aCL IgG) or MPL (aCL IgM) according to the standard curve constructed in each test with 5 dilution points of the Harris/Sapporo standards. AB2GPI are quantified as U/ml. Only medium–high titers of aPL were considered positive. The criteria recommended by the International Society of Thrombosis and Hemostasis (ISTH) Scientific and Standardization Committee (ISTH) for the standardization of lupus anticoagulant/antiphospholipid antibodies (LA/APA) were applied for the characterization of LA [29–31].

Pregnancy morbidity was defined as follows:

- Obstetric Manifestations: (a) Sidney criteria [24]; (b) non-criteria obstetric morbidity related to APS: 1–2 early pregnancy losses (< 10 weeks), preterm birth (between 34 and 36 + 6 weeks), late preeclampsia (> 34 weeks), abruptio placentae, and unexplained in vitro fertilization failures (> 2) [32].
- Definitions: (a) Pregnancy loss: early pregnancy loss (< 10 weeks) and/or fetal death (> 10 weeks); (b) adverse pregnancy outcome (APO): early pregnancy loss, fetal death, preeclampsia, abruptio placentae, and preterm birth (< 37 weeks).
- aGAPSS Calculation: the adjusted GAPSS was calculated as previously described [19]. In brief, hypertension (1 point), dyslipidemia (3 points), aCL (5 points), AB2GPI (4 points), and LA (4 points). aGAPSS risk was stratified according to Radin et al. [20] as low risk (< 6 points), medium risk (6–11 points), and high risk (≥ 12 points).

### Statistical Analysis

Results were expressed as numbers (percentage), mean ± standard deviation (SD), or median and interquartile range (IQR), as appropriate. Student’s *t*-test or Mann–Whitney *U*-test or one-way ANOVA were used to compare quantitative variables and chi-squared or Fisher test, to compare categorical data. A two-tailed *p*-value < 0.05 was considered statistically significant in all the calculations.

### Risk Prediction in Obstetric Antiphospholipid Syndrome: a Systematic Review of the Literature

**Fig. 1:**
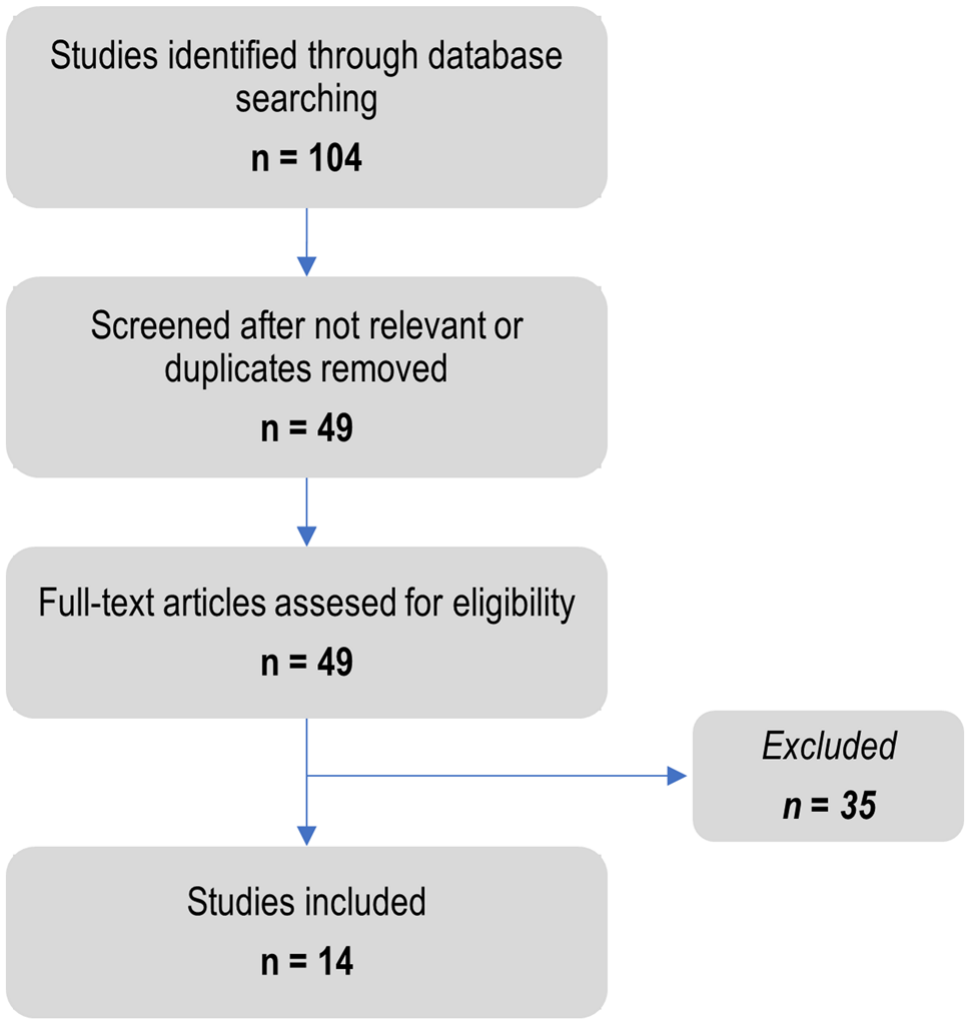
Flow diagram of the study selection process

A comprehensive literature search was conducted in PubMed and Embase. Electronic searches were supplemented by manual analysis of reference lists and reviews (up to October 2021). We used the following MeSH terms and keywords for searching PubMed: “antiphospholipid syndrome and GAPSS,” “antiphospholipid syndrome and APL-S,” “obstetric antiphospholipid syndrome and GAPSS,” “obstetric antiphospholipid syndrome and APL-S,” and “obstetric antiphospholipid syndrome and score.” Studies that included patients with obstetric APS and any predictive score were reviewed as shown in the flow chart (Fig. 1). Information was collected on study design, study sample, characteristics of the study population, and main results.

## Results

### General Features of the Study Cohort

During the study period, 137 consecutive patients with aPL fulfilled the inclusion and exclusion criteria. The main characteristics of the study cohort, their serological profile, and standard treatment are shown in Table 1. The mean age of the overall study group was 33.5 ± 45.5 years, and the patients were followed for more than 12 years (135.1 ± 89.9 months).

**Table 1:** Demographic characteristics, cardiovascular risk factors, main comorbidities, serological groups, and treatment in the different study groups

	**Primary APS (*N* = 65)**	**Non-criteria obstetric morbidity (*N* = 61)**	**Asymptomatic carriers (*N* = 11)**
Age (*yrs, mean* ± *SD)*	33.6 ± 4.8	33.3 ± 6.1	33.3 ± 5.7
Follow-up, *months (median, (95% IQR))*	121 (82.2–205.7)	96 (35.5–202) *	192 (74–254) *
Family history of thrombosis *(%)*	12.3	6.6	0
**Cardiovascular risk factors *(%)***	61.5	45.9	45.5
Obesity	21.7	10.9	0
Smoking	47.7	34.4	36.4
High blood pressure	9.2	9.8	9.1
Diabetes	3.1	1.6	0
Dyslipidemia	6.2	4.9	9.1
**Comorbidities *(%)***			
Inherited thrombophilia	18.9	8.9	10
Thyroid disease	12.3	13.1	0
Obstetric comorbidity	10.8	14.8	18.2
**Serological groups *(%)***			
Double/Triple +	30.8	27.9	54.5
LA +	18.5	9.8	18.2
aCL +	30.8	27.9	18.2
AB2GPI +	20	34.4	9.1
**Standard treatment *(%)***			
LDA	98.5	95.1	100
LWMH	76.9^**,***^	49.2^**^	36.4^***^
LDA + LWMH	76.9^**,***^	47.5^**^	36.4^***^
**aGAPSS** *(median, [95% IQR])*	6 (4–9)	5 (4–9)	8 (5–13)

*APS* antiphospholipid syndrome, *yrs* years, *SD* standard deviation, *IQR* interquartile range, *LA* lupus anticoagulant, *aCL* anticardiolipin antibodies, *AB2GPI* anti-b2- glycoprotein I, *LDA* low dose aspirin, *LWMH* low weight molecular heparin

^*^p < 0.05 (non-criteria obstetric morbidity versus asymptomatic carriers); ^**^p < 0.05 (primary APS versus non-criteria obstetric morbidity); ^***^p < 0.05 (primary APS versus asymptomatic carriers)

Patients in the primary APS group tended to have a higher frequency of family history of thrombosis, positive study for hereditary thrombophilia, and more cardiovascular risk factors, although these differences did not reach statistical significance. Overall, around one-third of the patients were double or triple positive for aPL, and interestingly, this rate tended to be higher in asymptomatic carriers (*p* = 0.1). AB2GPI antibodies were particularly frequent in the pregnancy-related morbidity group (*p* = 0.07).

After diagnosis, the majority of included subjects received standard treatment with low-dose aspirin (LDA) and/or low molecular weight heparin (LWMH) during pregnancies. Almost 80% of primary APS patients received combined therapy compared with 47.5% in the non-criteria group (*p* = 0.001) and 36.4% in the asymptomatic carriers (*p* = 0.001). No significant differences in the aGAPSS score between groups were found.

As shown in Table 2, aPL carriers had a lower number of pregnancies compared to primary APS (*p* < 0.0001) and non-criteria patients (*p* = 0.04). The primary APS group also had a higher number of pregnancies than the non-criteria group (*p* < 0.0001). Seventy-five percent of the patients had a live birth after treatment, and 37.2% had at least one APO. As expected, pregnancy loss and APO were significantly more frequent in the primary APS group (*p* < 0.05). More in detail, early abortion was significantly more frequent in the primary APS group compared to asymptomatic carriers (*p* = 0.03), and early abortion (*p* = 0.04), fetal death (*p* = 0.01), and preterm delivery (*p* = 0.01) compared with non-criteria patients.

**Table 2:** Obstetric outcome and main obstetric complications in the different groups after treatment

	**Primary APS (*N* = 65)**	**Non-criteria obstetric morbidity (*N* = 61)**	**Asymptomatic carriers (*N* = 11)**
**Pregnancy, number** *(median, (95% IQR))*	4 (3–5)^**,***^	3 (2–4)^**,*^	2 (1–3)^***,*^
**Live births *(%)***	76.6	73.8	72.7
**Pregnancy loss (%)**	35.4^***,**^	16.4^**^	0^***^
**Adverse pregnancy outcome *(%)***	55.4^***,**^	24.6^**^	0^***^
Abortion < 10 weeks	32.3^***,**^	16.4^**^	0^***^
Fetal death > 10 weeks	10.8^**^	0^**^	0
Preeclampsia/eclampsia	3.1	6.6	0
Preterm < 37 weeks	20^**^	4.9^**^	0
Abruptio placentae	0	1.6	0

*APS* antiphospholipid syndrome

^*^p < 0.05 (non-criteria obstetric morbidity versus asymptomatic carriers); ^**^p < 0.05 (primary APS versus non-criteria obstetric morbidity); ^***^p < 0.05 (primary APS versus asymptomatic carriers)

### Main Characteristics of the Patients According to the aGAPSS Risk

When patients were stratified according to aGAPSS values, 73 (53.3%) were categorized as low risk, 40 (29.2%) as medium risk, and 24 (17.5%) as high risk. The main characteristics of the patients according to the aGAPSS risk categories are shown in Table 3. Patients in the high-risk group were younger than those in the other two groups (*p* = 0.02 compared to medium risk and *p* = 0.004 compared to low-risk groups). Patients in the medium-risk group had more cardiovascular risk factors overall, although this difference was only significant for dyslipidemia compared to the low-risk group (*p* = 0.02). As expected, the majority of patients in the high-risk group carried a double/triple-positive aPL profile (*p* < 0.000), and patients in the low-risk group had mainly a single positive serological profile (*p* < 0.05). As shown in Table 3, the vast majority of patients in the three groups received LDA. LWMH and the combination of LDA and LWMH were more frequently used in the medium- (*p* = 0.02) and high-risk groups (*p* = 0.001) compared to the low-risk group. No significant differences in the number of pregnancies between the three aGAPSS groups (high: 3 [3, 4], medium 4 [2.25–4.75], or low risk 3 [2–5]) were found.

**Table 3:** Demographic characteristics, cardiovascular risk factors, and main comorbidities in the different study groups

	**aGAPSS < 6 (*N* = 73)**	**aGAPSS 6–11 (*N* = 40)**	**aGAPSS ≥ 12 (*N* = 24)**
Age *(yrs, mean* ± *SD)*	34.12 ± 5.09^**^	34.03 ± 5.6^*^	30.54 ± 5.48^*,**^
Follow-up, months *(median, (95% IQR))*	103 (60.5–202)	108 (65–182)	194.5 (59–232.5)
Family history of thrombosis *(%)*	9.6	10	4.2
**Cardiovascular risk factors *(%)***	47.9	62.5	54.2
Obesity	13.4	17.1	17.4
Smoking	41.1	42.5	37.5
High blood pressure	6.8	17.5	4.2
Diabetes	1.4	5	0
Dyslipidemia	1.4^***^	12.5^***^	8.3
**Comorbidities *(%)***			
Inherited thrombophilia	12.5	8.6	25
Thyroid disease	11	17.5	4.2
Obstetric comorbidity	20.5^***^	5^***^	4.2
**Serologic profile *(%)***			
Double/triple +	1.4^**,***^	47.5^*,***^	95.8^*,**^
LA +	23.3^**,***^	7.5^***^	0^**^
aCL +	38.4^**^	25^*^	4.2^*,**^
AB2GPI +	37^**^	20^*^	0^*,**^
**Standard treatment *(%)***			
LDA	94.5	100	100
LWMH	47.9^**,***^	70^***^	87.5^**^
LDA + LWMH	46.6^**,***^	70^***^	87.5^**^
**aGAPSS** *(median, (95% IQR))*	4 (4–5)	9 (7.25–9)	13 (12–13)

*APS* antiphospholipid syndrome, *yrs* years, *SD* standard deviation, *LA* lupus anticoagulant, *aCL* anticardiolipin antibodies, *AB2GPI* anti-b2- glycoprotein

^*^p < 0.05 (aGAPSS high-risk versus medium-risk); ^**^p < 0.05 (aGAPSS high-risk versus low-risk); ^***^p < 0.05 (aGAPSS medium-risk versus low-risk)

### Impact of aGAPSS Risk Stratification on the Obstetric Outcomes

**Fig. 2:**
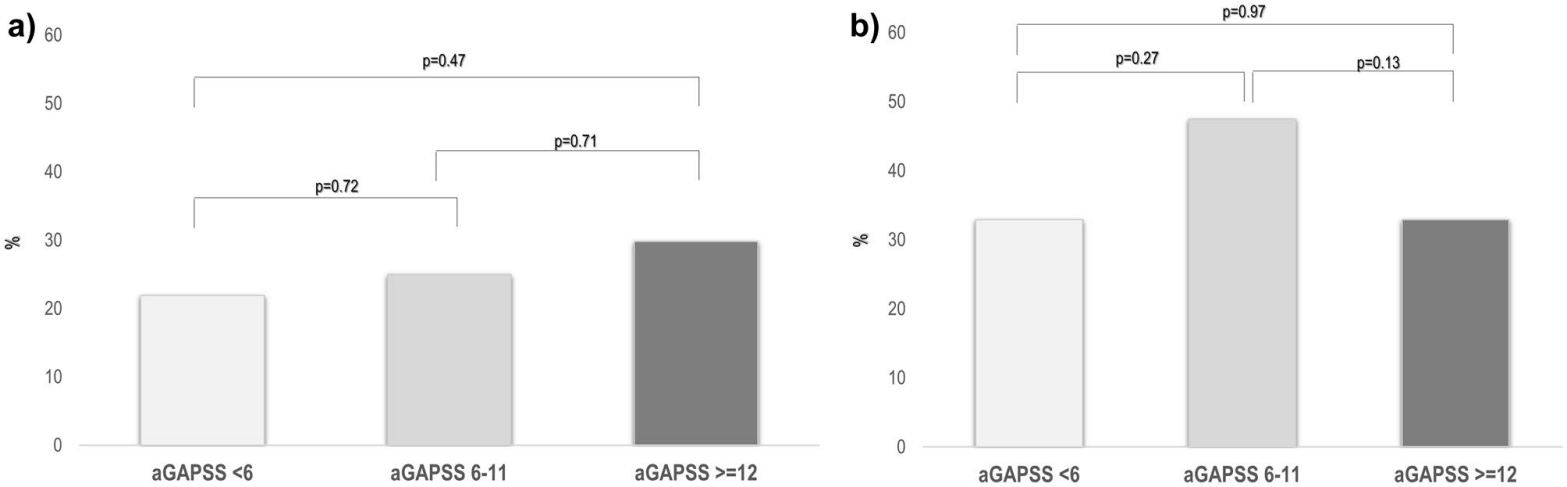
Pregnancy loss and adverse pregnancy outcomes (APO) in the three study groups according to aGAPSS categories after standard treatment. **a** Rates of patients with pregnancy loss expressed as percentages. **b** APO expressed as percentages in the three groups according to aGAPSS categories. aGAPSS risk was established according to Radin et al. [20]: low-risk (< 6 points), medium-risk (6–11 points), and high-risk (≥ 12 points)

As shown in Fig. 2a, there was a trend for an increase in pregnancy loss with the increase in aGAPSS risk. However, these differences were not statistically significant between groups. Furthermore, when we analyzed not only pregnancy loss but the overall obstetric complications included in the APO definition, we found no differences between low- and high-risk groups, and patients in the medium-risk category developed more frequently APO despite standard treatment (Fig. 2b).

### What Do We Learn from the Literature Review?

There is clear evidence of thrombosis recurrence and pregnancy complications in APS patients. In fact, up to 20–30% of the APS subjects present new clinical events, despite treatment [33]. Therefore, one of the most remarkable aspects of this syndrome is the appropriate stratification of refractory patients to initiate early treatment and avoid over-treating those with lower risk. In order to stratify the risk of developing clinical manifestations, several scores have been proposed (Table 4) [10–12, 14, 16, 20, 21, 34–40]. Two different algorithms were developed to stratify the thrombotic and obstetric risk: the antiphospholipid score (aPL-S) [12] and the Global Antiphospholipid Syndrome Score (GAPSS) [11]. APL-S was based only on the autoantibody profile [12]. However, thrombosis is a multifactorial condition, and cardiovascular risk factors (CVRF) such as smoking, hyperlipidemia, hypertension, and diabetes have been evaluated in APS patients and aPL carriers [41]. Therefore, the need to consider other relevant variables involved in the development of complications arises. Thus, Sciascia et al. [36] developed, in 2013, the GAPSS score that takes into account the combination of CVRF and aPL positivity profile. In the APS study cohort, only hyperlipidemia and hypertension remained as independent risk factors for developing any clinical complication in the multivariable analysis. The remaining CVRF did not show any significant difference, and therefore, they were not included in the score. In 2018, the same investigators [16] carried out a systematic review of the literature to assess the clinical utility of GAPSS and aGAPSS for risk stratification of any clinical manifestation of APS. They applied these algorithms to ten cohorts which included 2273 patients and found a statistically significant difference in both scores between patients who experience an arterial and/or venous thrombotic event (GAPSS, 10.6 ± 4.7 and aGAPSS, 7.6 ± 3.9) and those with obstetric morbidity (GAPSS, 8.8 ± 2.6 and aGAPSS, 6.7 ± 2.8). In 2020, Uludağ et al. [14] conducted a retrospective analysis to assess the effectiveness of aGAPSS to predict clinical manifestations in APS (both criteria and extra criteria). Ninety-eighth patients with APS and SLE were included and classified according to their clinical manifestations in vascular thrombosis, obstetric morbidity, or both. Significantly higher aGAPSS values were observed in the vascular thrombosis (*n* = 58) and vascular thrombosis plus obstetric morbidity (*n* = 29) groups, compared with those patients with obstetric morbidity only (*n* = 11) (10.6 ± 3.7 vs. 7.4 ± 2.9, *p* = 0.005, and 10.7 ± 4.0 vs. 7.4 ± 2.9).

**Table 4:** Summary of the studies devoted on clinical scores in obstetric APS

**Author, date**	**Study design**	**Study population**	**No. of patients**	**Objective**	**Main results**
Otomo et al. 2012 [12]	Retrospective	Patients with SAD	Group 1: *n* = 233 Group 2: *n* = 411	To define the aPL-S and evaluate its efficacy for the diagnosis of APS and predictive value for thrombosis	Higher values of the aPL-S in patients with thrombosis/obstetric morbidity vs. patients without signs of APS (*p* < 0.001). In the multivariate analysis, a level of aPL-S ≥ 30 is an independent risk factor for thrombosis
Sciascia et al. 2013 [35]	Retrospective	Patients with SLE	*N* = 211	Independent validation of the aPL-S	High values of aPL-S in patients with thrombosis/obstetric morbidity vs. patients without clinical signs of APS (*p* < 0.001). Positive predictive value of the aPL-S > 30, 36%, negative predictive value, 91%
Mondejar et al. 2014 [40]	Retrospective	Patients with APS	*N* = 116	To analyze the clinical performance of different aPL-S based on ELISA or CIA	Three aPL-Ss were calculated (ELISA, CIA, and CIA with D1 instead of β2GP1 IgG). The CIAs are comparable with the ELISAs for the detection of aPL antibodies. All three aPL-S were higher in individuals with thrombosis or pregnancy morbidity than in those without APS manifestations (*p* < 0.001)
Sciascia et al. 2013 [36]	Cross-sectional	Patients with SLE	*N* = 211	To develop and validate the GAPSS risk score	Patients were randomly divided into two groups: in the first group (*n* = 106), patients with high values of GAPSS are those with thrombosis and/or obstetric morbidity compared with those without clinical signs of APS. The second group (*n* = 105) was used to validate the GAPSS results obtained with the first group
Sciascia et al. 2014 [11]	Retrospective	Patients with primary APS	*N* = 62	To evaluate the clinical relevance of GAPSS in a cohort of primary APS patients	Higher GAPSS values were seen in patients who experienced thrombosis alone when compared with those with pregnancy loss alone (11.5 (S.D. 4.6) and 8.7 (S.D. 3.2), *p* = 0.04). Patients with both thrombosis and pregnancy loss showed higher GAPSS than those with pregnancy loss alone (12.5 (S.D. 4.6) vs. 8.7 (S.D. 3.2), *p* = 0.02). Patients with thrombotic recurrences showed higher GAPSS values when compared with those without recurrence (13.7 (S.D. 3.1) vs. 9.4 (S.D.3.9), *p* = 0.02). GAPSS values ≥ 11 seemed to have the best risk accuracy in terms of sensitivity and specificity
Oku et al. 2015 [34]	Retrospective	Patients with SAD	*N* = 282	Independent validation of GAPSS in a Japanese cohort of patients with autoimmune disease	Higher values of GAPSS were observed in patients who had experienced one or more of the APS manifestations compared with the patients without APS manifestations. Patients with a history of arterial and/or venous thrombosis showed higher GAPSS than patients without APS manifestations. Patients with a history of pregnancy morbidity failed to show a significant difference in GAPSS compared with patients without APS manifestations
Zuo et al. 2015 [37]	Retrospective	Patients with APS	*N* = 98	To evaluate the clinical relevance of aGAPSS in a Chinese APS cohort	Higher aGAPSS values were seen in patients who experienced thrombosis 9.4 ± 3.2, compared to those with pregnancy morbidity 6.7 ± 2.8, *p* = 0.0001. Patients who experienced both thrombosis and pregnancy morbidity showed higher mean aGAPSS when compared to those with pregnancy morbidity alone, but it was not statistically different (*p* = 0.087). There is no difference in aGAPSS score among patients who experienced thrombotic recurrence compared to those without recurrence
Sciascia et al. 2018 [16]	Systematic review	Patients with SAD	*N* = 2273	To evaluate the clinical utility of GAPSS and aGAPSS for risk stratification of any APS clinical manifestation	A statistically significant difference in the cumulative GAPSS and aGAPSS between patients that experienced an arterial and/or venous thrombotic event (cumulative mean GAPSS (s.d.) 10.6 (4.74) and aGAPSS 7.6 (3.95)) and patients with pregnancy morbidity (cumulative GAPSS 8.79 (2.59) and aGAPSS 6.7 (2.8)). The highest levels of GAPSS were found in patients that experienced arterial thrombosis (mean GAPSS 12.2 (5.2)) and patients that experienced any recurrences of clinical manifestations of APS (mean GAPSS 13.7 (3.1))
de Jesus et al. 2018 [48]	Retrospective	Women aPL carriers	*N* = 550	To evaluate the rate of thrombosis among Ob-APS	Younger age of Ob-APS, additional cardiovascular risk factors, superficial vein thrombosis, heart valve disease, and multiple aPL positivity increased the risk of the first thrombosis after PM. Women with thrombosis after PM had higher aGAPSS compared to those with Ob-APS alone ([median 11.5 [4–16] vs 9 [4–13], *p* = 0.0089])
Uludağ et al. 2021 [14]	Retrospective	Patients with APS and SLE	*N* = 98	To evaluate the validity of the aGAPSS in predicting clinical manifestations (criteria and extra-criteria) of APS	Significantly higher aGAPSS values were seen in VT and VT + PM groups when compared to PM group (10.6 ± 3.7 vs 7.4 ± 2.9, *p* = 0.005; 10.7 ± 4 vs 7.4 ± 2.9, *p* = 0.008, respectively). Higher aGAPPS values were also associated with recurrent thrombosis (11.6 ± 3.7 vs 9.9 ± 3.6, *p* = 0.04). Regarding extra-criteria manifestations, patients with livedo reticularis and APS nephropathy had significantly higher aGAPSS values (12.9 ± 3.4 vs 9.9 ± 3.7, *p* = 0.02; 12.4 ± 2.9 vs 10 ± 3.8, *p* = 0.04, respectively). The computed AUC demonstrated that aGAPSS values ≥ 10 had the best diagnostic accuracy for thrombosis
Liu et al. 2020 [39]	Retrospective	Patients with SAD	*N* = 522	To evaluate the clinical utility of anti-β2GPI-D1 IgG antibodies for stratifying the risk of thrombosis and/or PM in a cohort of Chinese patients with APS and assessed its correlation with the GAPSS	Anti-β2GPI-D1 antibodies were significantly higher in patients with triple aPL positivity than in those with double and single positive aPL (*p* < 0.001) and correlated well with the GAPSS (rho = 0.60, *p* < 0.001). Anti-β2GPI-D1 antibodies presented with a higher prevalence and higher titers in patients with late pregnancy morbidity (≥ 10 weeks) and thrombotic APS compared to those with early pregnancy (< 10 weeks) morbidity. Higher anti-β2GP1-D1 antibodies titers effectively distinguished APS from other autoimmune diseases
Radin et al. 2020 [20]	Retrospective	Women with aPL ever pregnant treated with SoC therapy	*N* = 143	To investigate the individual clinical response to SoC in women with aPL after stratifying them according to their GAPSS	When considering patients who ever experienced any PM while treated with SoC, all patients in the high-risk group experienced PM, while patients in the medium group had a significantly higher rate of PM when compared to the low-risk group [29 (43.9%) patients vs.11 (15.3%), respectively; *p* < 0.001]. Patients in the high-risk group had significantly lower live birth rates when compared to the other groups (11 (40.7%) live births vs. 100 (62.1%) and 137 (82.5%), respectively; *p* < 0.05). Patients with the medium-risk group also had significantly lower live birth rates when compared to the lower risk group (*p* < 0.001)
Schreiber et al. 2021 [21]	Retrospective	Women ever pregnant with SLE and APS	*N* = 143	To validate the GAPSS in a cohort of women with SLE and aPL	Women with any placental medicated complication (fetal death, placental abruption, prematurity, pre-eclampsia, or IUGR) have significantly higher GAPSS values. Patients with three or more consecutive early miscarriages (< 10 weeks), fetal death, miscarriage < 10 weeks' gestation, premature birth (< 34 weeks), pre-eclampsia (< 34 weeks), stillbirth, and placental infarction had significantly higher GAPSS values compared to those without previous pregnancy complications. The odds ratio of having any pregnancy morbidity when having a GAPSS value ≥ 8 was 20 compared to those with a GAPSS of ≤ 1 (*p* < 0.001)
Pregnolato et al. 2021 [10]	Retrospective	Women with APS and SAD and negative aPL	*N* = 381	To investigate the impact of aPL positivity fulfilling classification criteria and at titers lower than thresholds considered by classification criteria on PM and assesses the effectiveness of treatment in reducing the probability of PM (PPM)	PPM was further stratified upon the aPL tests: aCL IgG/IgM and anti-β2GPI IgM, alone or combined, do not affect the basal risks of PPM, an increase occurs in case of positive LA or anti-β2GPI IgG. LDASA significantly affects PPM exclusively in women with low titer aPL without anti-β2GPI IgG. LDASA + LMWH combination significantly reduces PPM in all women with low titer aPL and women with criteria aPL, except those carrying LA and anti-β2GPI IgG. In this group, the addition of HCQ further reduces PPM, although not significantly

*APS* anti-phospholipid syndrome, *SAD* systemic autoimmune disease, *aPL-S* anti-phospholipid score, *GAPSS* Global Antiphospholipid Syndrome Score, *aGAPSS* adjusted Global Antiphospholipid Syndrome Score, *SLE* systemic lupus erythematosus, *APS* anti-phospholipid syndrome, *aPL* anti-phospholipid antibodies, *aCL* anti-cardiolipin, *β2GPI* β2-glycoprotein I, *ELISA* enzyme-linked immunoassay, *CIA* chemiluminescent immunoassay, *LA* lupus anticoagulant, *PM* pregnancy morbidity, *IUGR* intrauterine growth restriction, *LDASA* low-dose aspirin, *LMWH* low molecular weight heparin, *HCQ* hydroxychloroquine, *Ob-APS* obstetric anti-phospholipid syndrome, *β2GPI-D1* β2-glycoprotein I domain 1, *SoC* standard of care, *AUC* area under the curve

After the systematic review of the literature, three retrospective studies of GAPSS in obstetric APS were identified. In 2018, de Jesus et al. [38] performed a retrospective analysis from an APS multicenter database. Of 126 patients with obstetric APS, 74 presented thrombosis, and 47 of them developed thrombosis after the initial obstetric complication during a mean follow-up of 8 years. Younger age at the time of APS diagnosis, the presence of additional CVRF (smoking, hypertension, or hyperlipidemia), venous thrombosis, valvular heart disease, and multiple aPL positivity increased the risk for a first thrombotic event after the obstetric complication. Women who suffered a thrombotic event after the obstetric complication had a higher aGAPSS than women with obstetric APS alone (median, 11.5 (4–16) vs. 9 (4–13); *p* = 0.0089). They concluded that the aGAPSS is a valuable tool to improve the risk stratification in women with aPL.

More recently, Radin et al. [20] investigated the individual clinical response to standard therapy in women with APS after stratifying by GAPSS. One hundred and thirty-three women with aPL (352 pregnancies) treated with standard therapy were included. They were grouped, according to their GAPSS value, into low (< 6, *n* = 72), medium (6–11, *n* = 66), and high risk (> 12, *n* = 5). The live birth rate was 70.5% (248 out of 352 pregnancies). When they analyzed the number of pregnancies in the three groups, women with high risk had a significantly lower live birth rate than the other groups (11 (40.7%) live births vs. 100 (62.1%) and 137 (82.5%), respectively; *p* < 0.05).

In the same year, Schreiber et al. [21] conducted a study to validate GAPPS in a cohort of 143 women with pregnancy history and diagnosed with SLE. Patients with three or more early consecutive miscarriages (< 10 weeks), fetal death, one spontaneous miscarriage before 10 weeks of gestation, preterm birth (< 34 weeks), preeclampsia, and placental infarction had significantly higher GAPSS values than those without previous pregnancy complications. The odds ratio of having obstetric complications with GAPSS values > 8 was 20, compared with those with GAPSS < 1 (*p* < 0.001).

Regarding the obstetric complications in APS, it is worthy to note that the GAPPS/aGAPPS algorithms have been validated in a few studies, including patients with obstetric APS and those who did not strictly match the disease criteria, becoming part of the “non-criteria obstetric APS.” Within this large group of patients, some of them meet the clinical but not the serological criteria “(inconclusive serologic APS”), although they could benefit from preventive therapy. This supports the need to modify the existing risk scores, adding the high aPL titers and the low ones, including many patients who fit this feature but are currently excluded. Taking this into account, a new algorithm called EUREKA was developed to stratify the probability of obstetric complications in APS patients with different aPL titers and evaluate the effectiveness of the therapy based on the aPL profile [10]. They conducted a retrospective study in 381 women with 155 aPL carriers and 226 having some autoimmune disease but negative aPL. This study aimed to investigate the impact of aPL positivity in the development of obstetric complications, both at medium–high titers (included in the classification criteria for APS) and at low titers (non-conventional criteria). Besides, the authors analyzed the efficacy of the therapy with acetylsalicylic acid (ASA), low molecular weight heparin (LMWH), and hydroxychloroquine (HCQ) to reduce the likelihood of development obstetric complications.

Regarding the impact of the aPL in obstetric morbidity, the probability of developing obstetric complications in women with autoimmune disease and negative aPL was 39%. Meanwhile, in those with positive aPL, this probability was 64% with low titers and 68% with high titers with a particularly higher risk in those with LA and/or IgG anti-β2GPI positivity (86% and 76%, respectively). Concerning the efficacy of the therapy, those subjects with low aPL titers (without IgG anti-β2GPI) benefited from ASA monotherapy and in association with LMWH or from triple therapy with HCQ. However, although not significantly, LA and IgG anti-β2GPI carriers (high risk) the triple therapy reduced the probability of obstetric complications.

## Discussion

In the present study, we evaluate the utility of the aGAPSS score to identify the response to treatment in aPL carriers during subsequent pregnancies. As shown here, the aGAPSS score that includes cardiovascular risk factors and the aPL profile does not allow stratifying the patients at higher risk of obstetric complications despite standard treatment.

To the best of our knowledge, only one recent study reported the possible usefulness of GAPSS as a tool to stratify the risk of obstetric complications in pregnant subjects with aPL [20]. In that study, which mainly included patients with SLE, it was suggested that GAPSS could be a useful tool to stratify the response to standard treatment. Among the advantages of this type of approach, the authors suggested the possibility of adjusting the treatment guidelines for high-risk groups, its potential utility in the development of future therapeutic schemes, and the benefit of having, in daily clinical practice, a simple tool without additional cost. Besides, it would allow the identification of those patients who present only obstetric symptoms and are at risk of developing future thrombotic complications. As shown here, we cannot confirm the utility of the aGAPSS in our study population. However, as the frequency of thrombotic events during the follow-up was extremely low in our cohort, we could not assess the last possibility.

Several possible explanations could justify our results. First of all, the aGAPSS includes traditional cardiovascular risk factors such as hypertension and dyslipidemia. Although highly relevant in developing thrombotic processes, given their low frequency in the population of women of childbearing age, they may provide little discriminatory value over other factors more directly related to obstetric outcomes. Thus, hypertension has a clear impact on the obstetric prognosis of pregnant women [42], whereas dyslipidemia, which has a high score on the aGAPSS, does not have such a defined role during pregnancy [43]. On the other hand, while there is some consensus that LA is the main antibody related to obstetric morbidity in patients with aPL [44, 45], LA score in the aGAPSS is lower than aCL antibodies. Furthermore, the presence of double/triple positivity, which is associated with a higher frequency of clinical APS [25, 46], is not considered as a differential risk factor in the aGAPSS. Finally, and although unlikely, we cannot exclude the possibility that using aGAPSS instead of GAPSS, which includes antiphosphatidyl serine/prothrombin antibodies, may have influenced our final results.

**Fig. 3:**
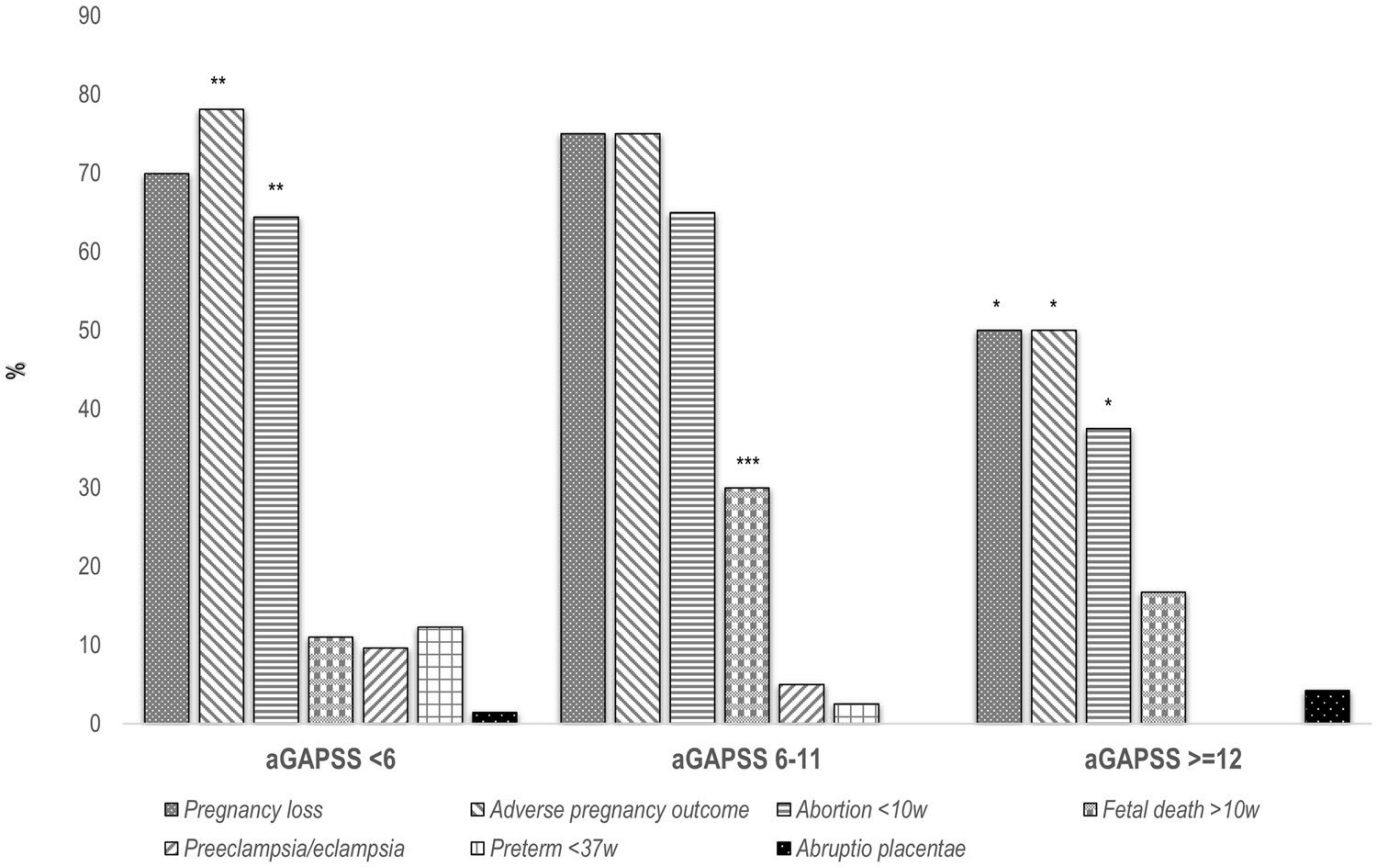
Pregnancy loss and adverse pregnancy outcomes (APO) in the three groups according to aGAPSS categories before treatment. Rates of patients with pregnancy loss and APO were expressed as percentages in the three groups according to aGAPSS categories before standard treatment. aGAPSS risk was established according to Radin et al. [20]: low-risk (< 6 points), medium-risk (6–11 points), and high-risk (≥ 12 points). *p < 0.05 (aGAPSS ≥ 12 vs. aGAPSS 6–11); **p < 0.05 (aGAPSS ≥ 12 vs. aGAPSS < 6); ***p < 0.05 (aGAPSS 6–11 vs. aGAPSS < 6)

When assessing the risk of obstetric morbidity, especially pregnancy loss, regardless of the presence or absence of aPL, two are key factors in further pregnancies: age and previous obstetric history [47]. In this regard, patients in the high-risk group were significantly younger than patients in the medium and low-risk categories. Furthermore, as shown in Fig. 3, patients in the medium- and low-risk groups had a worse previous obstetric history concerning both pregnancy loss and APO. Therefore, the possible role of the aGAPSS to predict the risk of further obstetric complications was modulated by the two main factors related to obstetric outcomes. For these reasons, and regardless of the positivity of aPL, future scores that look to assess the risk of future complications and the impact of the different treatments on women with aPL should specifically consider at least these two variables.

Our study has certain limitations. First are those inherent to a retrospective design. Besides, it is carried out in a single center and a multidisciplinary unit specifically devoted to the treatment of obstetric complications in patients with autoimmune diseases. This means that the results cannot be extrapolated to other populations and probably to the care of pregnant patients outside highly specialized units. Although the group of asymptomatic carriers included in our cohort is small, they present similar demographic characteristics to the other two groups analyzed.

Furthermore, the vast majority of these asymptomatic carriers present a high-risk serological profile, with almost three-quarters of them carrying LA or double/triple positivity. The possibility that these patients would develop clinical manifestations during a longer follow-up is unlikely since they have been followed up for a very long period, and in our experience, asymptomatic carriers tend to develop clinical manifestations earlier [41]. Finally, considering whether patients who do not meet clinical criteria for the disease or even if patients with a high-risk serological profile should be treated similarly to patients with APS remains a subject of intense debate [48, 49], although the obstetric outcomes are similar when treated [50].

We consider that our study has certain advantages over previous ones. Firstly, these studies have been carried out in patients with aPL associated with other autoimmune diseases, mainly SLE, whereas those patients have been excluded from our study. Thus, we could analyze a more homogeneous population of patients belonging to the clinical spectrum of APS. Secondly, the present cohort represents the whole spectrum of patients with aPL. It ranges from asymptomatic carriers to patients with primary APS, defined according to the classification criteria [24], and includes patients with aPL who present obstetric manifestations not included in these criteria but represent a very relevant subgroup in routine clinical practice. Another advantage of our study is that in addition to the cardiovascular risk factors and the serological profile, we have also included other comorbidities that could influence the overall obstetric prognosis [51–54]. Although we did not find significant differences in these comorbidities when we analyzed the groups of aPL carriers, after stratifying by aGAPSS, the patients included in the low-risk group did present a higher proportion of obstetric comorbidities (*p* = 0.027 compared with the medium risk and *p* = 0.11 with high-the risk group). However, we consider that these differences have not contributed significantly to our results.

After an extensive revision (Table 5), it seems that the GAPSS/aGAPSS might be superior to the aPL-S. The findings that support this fact are the addition of CVRF, the greater scientific evidence of their clinical utility in thrombotic APS compared with the aPL-S (19 studies based on GAPSS, and 5 about aPL-S), the addition of extra criteria manifestations that allow better detection of associated complications, and finally, its clinical simplicity. However, despite the GAPSS/aGAPSS benefits, there are some pending studies of validation in conventional and non-conventional obstetric APS. As our study points out, further studies are needed, and probably, the addition of other factors related to the pregnancy should be considered. In an attempt to resolve part of this gap, Pregnolato et al. [10] developed the EUREKA algorithm, including the low aPL titers, which means that those women who were excluded from the diagnostic of APS, and therefore, without treatment, will be considered. Since there is only one retrospective study on this issue, further validation of the EUREKA algorithm will be necessary.

**Table 5:** Usefulness and comparison of clinical scores

	**aPL-S**	**GAPSS/aGAPSS**	**EUREKA**
**Usefulness**	APS diagnostic tool and predictive marker of thrombosis in autoimmune diseases	Risk stratification tool and predictor of thrombosis in APS and other associated autoimmune diseases	The first risk stratification tool developed explicitly in the context of aPL-related pregnancy complications
**Comparative advantages**	Possibility of using the partial aPL-S scale (exclusively assesses aPL included in the revised Sapporo criteria)	• Combines the independent CVRF (hyperlipidemia and arterial hypertension) and the aPL positivity profile • More significant scientific evidence of its usefulness in thrombosis APS • Greater simplicity: requires fewer aPL determination tests • The addition of obesity, smoking, and diabetes in aGAPSS allows a higher rate of CVD detection in subjects with APS (need for validation) • Possibility of using aGAPSS, which excludes aPS/PT determination	• Includes aPL at low titers (extra criteria) relevant in the obstetric manifestations but not in the thrombotic APS • Considers different aPL tests, which allows the identification of two risk categories for aPL at low titers and four for aPL at medium–high titers • Association between each of the aPL tests and obstetric morbidity • Predicts response to treatment in each clinical situation

*APS* anti-phospholipid syndrome, *aPL-S* anti-phospholipid score, *aPL* anti-phospholipid antibodies, *GAPSS* Global Antiphospholipid Syndrome Score, *aGAPSS* adjusted Global Antiphospholipid Syndrome Score, *CVRF* cardiovascular risk factors, *CVD* cardiovascular disease, *aPS/PT* anti-phosphatidylserine/prothrombin

In summary, in the present study, including aPL carriers without other autoimmune diseases, the aGAPSS does not seem to be a valuable tool to identify patients at risk for obstetric complications despite treatment. In these patients with gestational desire, in addition to the aPL profile, other pregnancy-specific factors, such as age or previous obstetric history, should be considered.

## Data Availability

Due to research still being conducted on the project in our research group, full data are not available. Additional data is available upon reasonable request to the corresponding author.
